# PET/MRI for Preoperative Planning in Patients with Soft Tissue Sarcoma: A Technical Report of Two Patients

**DOI:** 10.1155/2013/791078

**Published:** 2013-12-04

**Authors:** Annika Loft, Karl Erik Jensen, Johan Löfgren, Søren Daugaard, Michael M. Petersen

**Affiliations:** ^1^Department of Clinical Physiology, Nuclear Medicine & PET, Rigshospitalet, University of Copenhagen, Blegdamsvej 9, DK-2100 Copenhagen, Denmark; ^2^Department of Radiology, Rigshospitalet, University of Copenhagen, Blegdamsvej 9, DK-2100 Copenhagen, Denmark; ^3^Department of Pathology, Rigshospitalet, University of Copenhagen, Blegdamsvej 9, DK-2100 Copenhagen, Denmark; ^4^Department of Orthopedic Surgery, Rigshospitalet, University Hospital of Copenhagen, Blegdamsvej 9, DK-2100 Copenhagen, Denmark

## Abstract

Clinical positron emission tomography (PET)/magnetic resonance imaging (MRI) acquisition protocols may improve the evaluation of soft tissue sarcomas (STS) prior to surgical planning. We examined two patients with lower extremity STS using a Siemens Biograph mMR PET/MRI scanner and the glucose analogue 18F-fluoro-deoxyglucose (FDG). We investigated clinically relevant tumor volumes and evaluated the relations to skeletal periosteum and nerve bundles. The patient scans suggest that FDG PET/MRI improved the edge detection, and invasion of tumor tissue into important adjacent anatomical structures can be evaluated. FDG PET/MRI also provided additional information compared to conventional Gadolinium enhanced MR imaging. The findings were proven by subsequent pathological examination of the resected tumor tissue. In the future, clinical FDG PET/MRI may be an important modality for preoperative planning, including radiation therapy planning in patients with STS.

## 1. Introduction

Soft tissue sarcomas (STS) comprise a heterogeneous group of mesenchymal derived tumors. Historically, the primary treatment for most STS is surgical excision with a wide margin, and amputation if a wide margin cannot be obtained [1]. However, over the past decades, it has been accepted by many surgeons to use also less mutilating limb-sparing operative procedures because when combined with external radiation therapy, marginal or even intralesional margins in STS surgery often provide an acceptable local tumor control [2, 3].

In the preoperative planning and workup of STS patients, Gadolinium enhanced magnetic resonance imaging (MRI) is the current gold standard. Although conventional MRI offers an excellent soft-tissue contrast and is the most versatile modality in musculoskeletal imaging, there are limitations concerning the exact definition of tumor infiltration into the peritumoral edema and the adjacent structures, which is essential for therapy planning. Moreover, oncologic therapy monitoring and follow-up after treatment remain challenging.

In Gadolinium enhanced MRI, edge detection is still affected by peritumoral inflammation and edema which leads to overestimation of tumor volume and extension. If major blood vessels, major nerve bundles, or bones are close to the tumor, it could lead to overestimation of invasive growth and hereby result in major surgical intervention or amputation. Hence, a more precise and specific imaging method may reduce the morbidity following sarcoma surgery.

The new hybrid positron emission tomograph combined with a 3 Tesla (T) MRI scanner (PET/MRI) provides the possibility for simultaneous PET/MRI and combines the highly sensitive molecular imaging capability of PET with the superior soft-tissue contrast of MR imaging [4, 5].

The purpose of our study was to evaluate the tumor delineation by use of clinical PET/MRI performed in two adult patients with limb STS and compare the results with subsequent pathological data from the resected tumor.

## 2. Material and Methods

The patient examinations were performed using a new hybrid PET/MRI system (Biograph mMR, Siemens) which allows for simultaneous acquisition of PET and MR data. The system consists of an MR-compatible whole-body-PET system (Biograph, Siemens) inserted into a modified whole-body MR scanner (Trio, Siemens) at a field strength of 3 T.

The technical specifications have previously been described in detail [6]. The PET scanner has an axial field of view of 26 cm. A standard transmit/receive body coil was used for excitation and a receive-only surface coil was used for signal reception.

After gradient echo localizers, a transverse Dixon sequence for attenuation correction was performed. This was followed by (1) sagittal short inversion time inversion recovery (STIR) sequence, (2) axial T1-weighted spin-echo (SE) sequence, and (3) axial T2 fast SE sequence with pulsed fat saturation.

After an intravenous injection of FDG 4 MBq/kg body weight, the patient rested for 60 minutes before a diagnostic whole-body PET/CT scan for staging purposes was performed in a dedicated Siemens Biograph 64 PET/CT scanner. Immediately after the PET/CT scan, the PET/MRI scan was performed. The PET acquisition time of the following PET/MRI was 5 minutes per bed position, due to the interval between injection and scanning.

PET and MRI data acquisition was done simultaneously. The PET data was reconstructed with AW-OSEM (3 iterations, 21 subsets, and 4 mm Gaussian filter) on 344 × 344 matrices without PSF. The fused PET/MR images were viewed and interpreted on the Siemens Syngovia system.

The pathological specimens were routinely processed (formalin fixation and paraffin embedding). In Case 1, however, the amputated specimen was initially frozen (−20°C) and then sectioned on a band saw to demonstrate the tumor's relation to the bone. In Case 2, macrosections (4 × 5 cm) were taken from the area of interest at the site of the sciatic nerve (marked by the surgeon).

### 2.1. Patients

**Figure 1:**
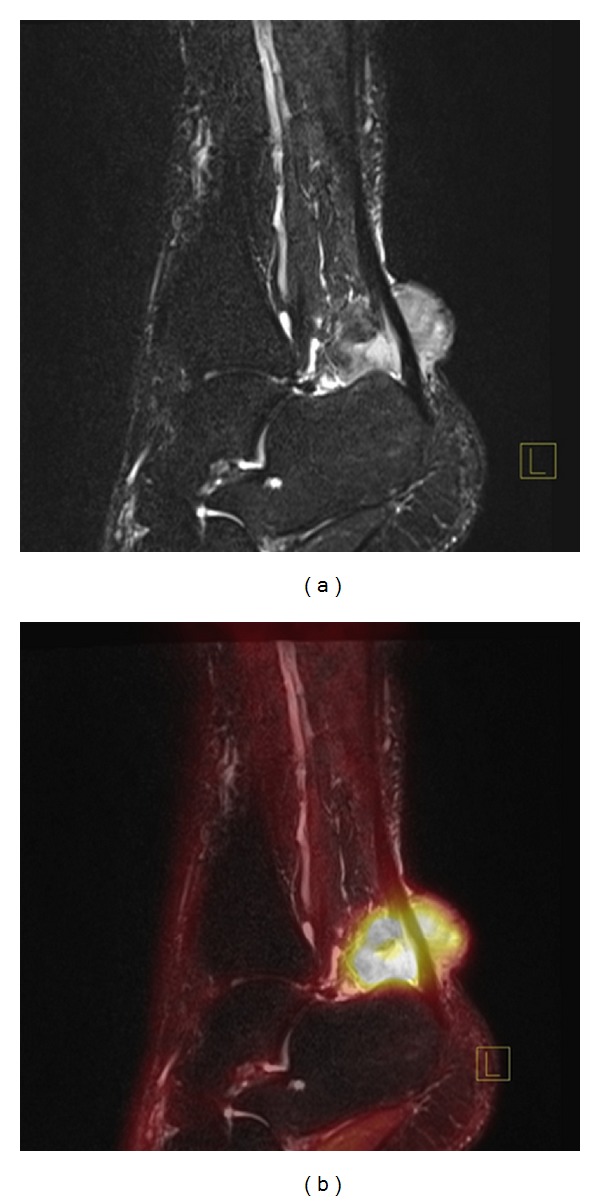
T1-weighted postgadolinium MRI shows a sagittal section of a clear cell sarcoma surrounding the Achilles tendon (Case 1), and tumor ingrowth to the calcaneus cannot be excluded (a). Fused FDG-PET and MRI show no tumor involvement of the bone (b).

**Figure 2:**
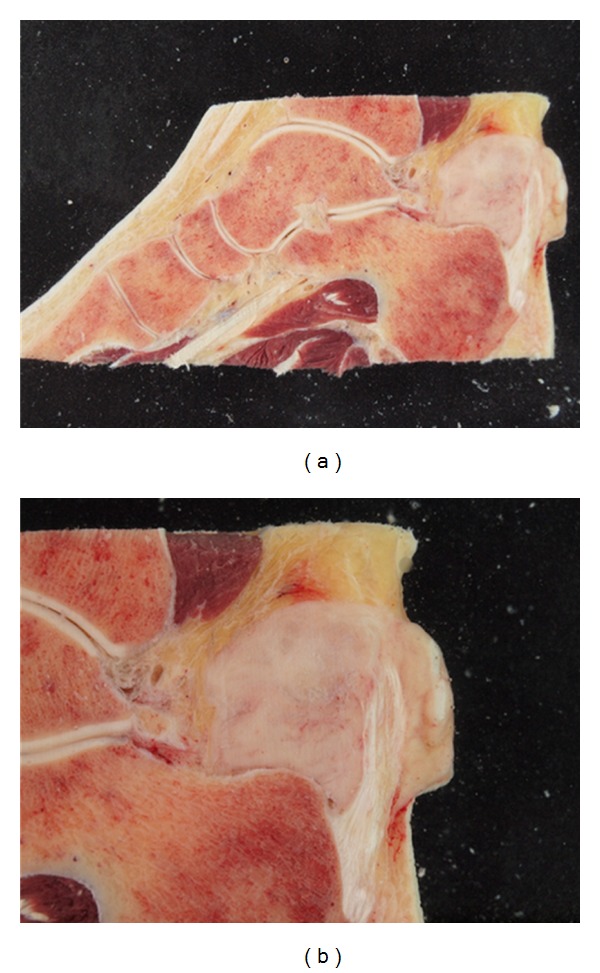
Sagittal sections of the ankle region of the amputated specimen (Case 1) showing the (clear cell) sarcoma involving the Achilles tendon with a close relation to the calcaneus but without macroscopically visible invasion of the bone.

**Figure 3:**
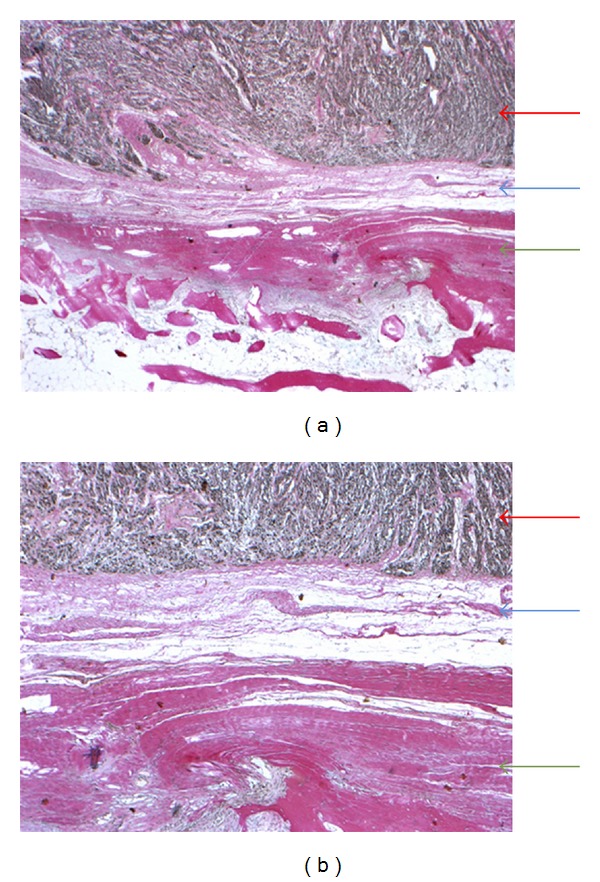
Histology Case 1. Alcian-van Gieson stain in the original magnification ×20 (a) and ×40 (b) showing the clear cell sarcoma (red arrow), its pseudocapsule (blue arrow), and the periosteum (green arrow). There is no penetration of the pseudocapsule and no invasion of the bone.

*Case 1*. A 50-year-old adipose (weight 157 kg) male was referred with a needle-biopsy verified grade 2 clear cell sarcoma and no signs of disseminated disease on a FDG PET/CT performed at a local hospital. The tumor was located in the left lower limb around the Achilles tendon with contact to the periosteal tissue of the calcaneus, and MRI could not exclude tumor invasion into the calcaneus (Figure 1). Limb-sparing surgery with tumor excision and reconstruction of the Achilles tendon and skin defect with a free flap was considered, and therefore a FDG PET/MRI was performed with the aim of evaluating potential tumor ingrowth to the bone. PET/MRI provided excellent metabolic information (SUVmean = 16, SUVmax = 21), suggesting no involvement of the bone (Figure 1). However, because of patient-related reasons (age and overweight), the patient and the surgeon settled for radical surgery with an amputation. Postoperative pathology examination revealed that the surgical margin was free of tumor and no ingrowth of tumor to the bone could be documented (Figures 2 and 3), thus matching the PET/MRI information obtained prior to surgery.

**Figure 4:**
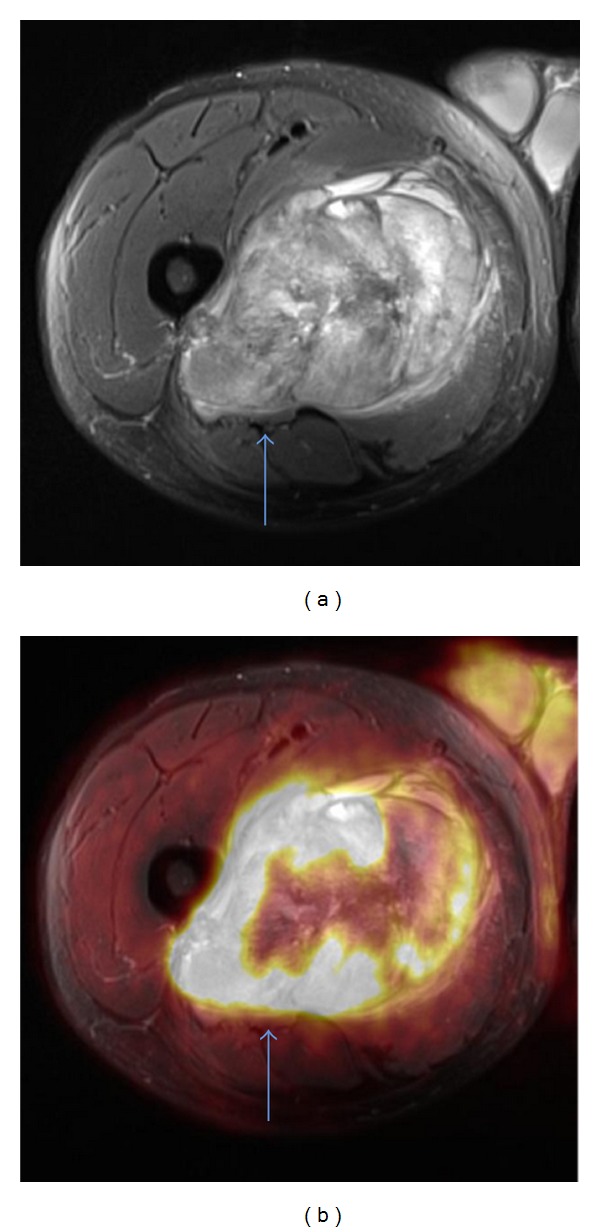
T1-weighted postgadolinium MRI shows a high-grade pleomorphic liposarcoma in the adductor muscles of the right femur (a) and tumor invasion into the sciatic nerve could not be elucidated (Case 2). Fused FDG-PET and MRI show no tumor involvement of the sciatic nerve (b). Sciatic nerve is marked with a blue arrow.

**Figure 5:**
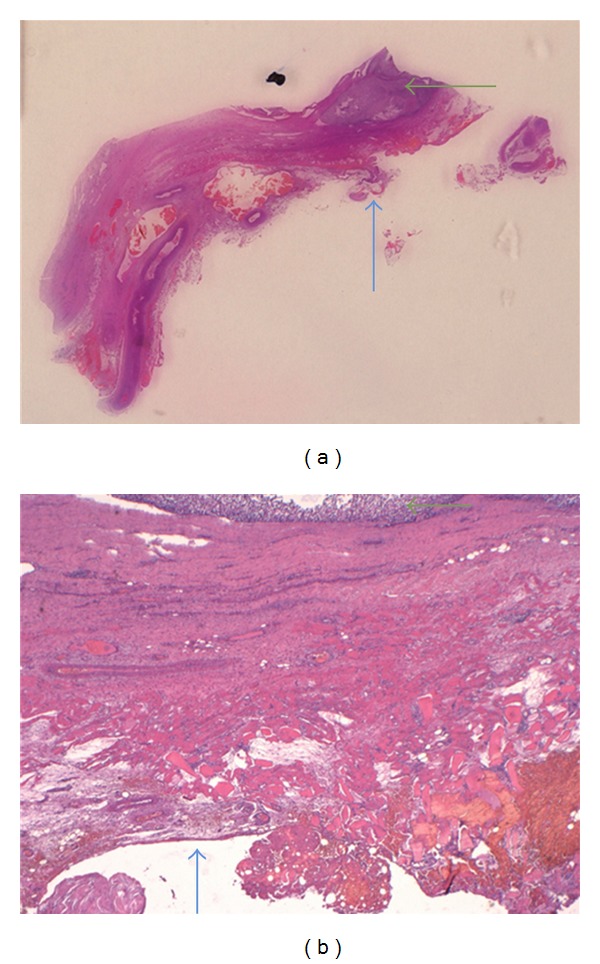
Histology Case 2. HE stained macrosection (picture 4 cm wide) in original magnification (a) and ×20 (b) showing the bed where the sciatic nerve was located (blue arrow) in close proximity to the tumor (green arrow).

*Case 2*. A 53-year-old male was referred from Greenland for further diagnostics because of a large soft tissue mass of the adductor muscles of the right femur initially verified on a CT scan. A MRI and a core needle biopsy were performed, and the histological diagnosis was a grade 3 pleomorphic liposarcoma. From the MRI, tumor invasion to the sciatic nerve could not be elucidated and therefore it was decided to perform a PET/MRI following the initial diagnostic FDG PET/CT scan (using the same FDG dose). FDG PET/CT showed no signs of distant metastases and the PET/MRI showed both the sciatic nerve and the femoral vessels to be free of FDG PET positive (SUVmean = 14, SUVmax = 18) tumor ingrowth (Figure 4). The day before surgery, embolization of tumor vessels from the deep femoral artery to the tumor was performed by a specialist in cardiovascular radiology. A marginal resection of the tumor was performed sparing the femoral vessels and the sciatic nerve. The pathologists report documented that the tumor was removed with at least a marginal margin and no tumor cells were found at the margins related to these structures (Figure 5). The treatment was completed with external radiation therapy.

## 3. Discussion

The conventional MRI quality was diagnostic in both cases, but the added metabolic information from FDG PET in the PET/MRI provided valuable information in both patients regarding possible tumor invasion into adjacent bone or nerves. In tumors with great variability in signal intensity and ill-defined edges, PET/MRI may significantly improve the delineation. Since PET and MRI information is acquired simultaneously, motion artifacts and partial volume effects are eliminated. Hence, PET/MRI represents significant improvement compared to existing coregistration of PET with MRI data.

In the presented evaluation of PET/MRI image data obtained in 2 STS patients, we did not observe any image quality reduction compared to conventional MRI and PET scanning.

While image-guided intensity modulated radiotherapy with tight margins is the norm in other disease sites such as brain tumors, the implementation of advanced technology has somewhat lagged in STS. When treatment techniques are simple and margins are wide, small differences in tumor delineation may be less consequential. However, as clinical target volumes shrink and inverse-planned radiation becomes more prevalent in the treatment of STS, more attention will be needed in the quality assurance of contouring before radiation treatment.

Other PET tracers than FDG may provide additional information suitable for a combined PET/MRI scanner; future investigations are expected.

## 4. Conclusion

Our results indicate that combined PET/MRI compared to conventional Gadolinium enhanced MRI could be a clinically relevant imaging technique that can change the preoperative planning prior to limb sparing surgery in patients with STS.

## References

[1] Cantin J, McNeer GP, Chu FC, Booher RJ (1968). The problem of local recurrence after treatment of soft tissue sarcoma. *Annals of Surgery*.

[2] Trovik CS, Bauer HCF, Berlin Ö (2001). Local recurrence of deep-seated, high-grade, soft tissue sarcoma: 459 Patients from the Scandinavian Sarcoma Group Register. *Acta Orthopaedica Scandinavica*.

[3] Jebsen NL, Trovik CS, Bauer HCF (2008). Radiotherapy to improve local control regardless of surgical margin and malignancy grade in extremity and trunk wall soft tissue sarcoma: a Scandinavian sarcoma group study. *International Journal of Radiation Oncology Biology Physics*.

[4] Pichler BJ, Kolb A, Nägele T, Schlemmer H-P (2010). PET/MRI: paving the way for the next generation of clinical multimodality imaging applications. *Journal of Nuclear Medicine*.

[5] Ratib O, Beyer T (2011). Whole-body hybrid PET/MRI: ready for clinical use?. *European Journal of Nuclear Medicine and Molecular Imaging*.

[6] Delso G, Fürst S, Jakoby B (2011). Performance measurements of the siemens mMR integrated whole-body PET/MR scanner. *Journal of Nuclear Medicine*.

